# Effects of Fibrin Clot Inhibitors and Statins on the Intravesical Bacille Calmette–Guérin Therapy for Bladder Cancer: A Systematic Review and Meta-Analysis

**DOI:** 10.3389/fonc.2021.614041

**Published:** 2021-06-21

**Authors:** Zhiyong Cai, Jiao Hu, Belaydi Othmane, Huihuang Li, Dongxu Qiu, Zhenglin Yi, Jinbo Chen, Xiongbing Zu

**Affiliations:** Department of Urology, Xiangya Hospital, Central South University, Changsha, China

**Keywords:** statins, fibrin clot inhibitors, Bacille Calmette–Guérin, bladder cancer, prognosis

## Abstract

**Objective:**

To assess the effect of fibrin clot inhibitors (aspirin, clopidogrel, and warfarin) and statins on intravesical BCG therapy.

**Method:**

A systematic literature search was carried out through PubMed, Embase, and the Cochrane Central Search Library in March 2020. Accumulative analyses of odds ratios (ORs), hazard ratio (HR), and corresponding 95% confidence intervals (CIs) were performed. All analyses were performed by using Review Manager software version 5.3 and Stata 15.1.

**Results:**

Four cohort studies and nine case–control studies containing 3,451 patients were included. The pooled analysis indicated that statins (HR = 1.00; 95%CI, 0.82 to 1.22; *p* = 1.00) and fibrin clot inhibitors (HR = 1.01; 95%CI, 0.64 to 1.59; *p* = 0.98) did not affect the efficacy of BCG on recurrence-free survival. The cumulative analysis showed that statins (HR = 0.79; 95%CI, 0.41 to 1.49; *p* = 0.46) and fibrin clot inhibitors (HR = 1.62; 95%CI, 0.90 to 2.91; *p* = 0.11) did not affect the efficacy of BCG on progression-free survival. There were no differences on cancer-specific survival (HR = 1.68; 95%CI, 0.64 to 4.40; *p* = 0.29) and overall survival (HR = 1.13; 95%CI, 0.73 to 1.78; *p* = 0.58) after taking statins.

**Conclusion:**

The present study shows that the application of fibrin clot inhibitors and statins do not influence the efficacy of BCG on oncological prognosis. Consequently, we do not need to stop using them or change to other drugs during intravesical BCG treatment. However, large-scale multi-center prospective research is still needed to confirm the above conclusions.

## Introduction

Bladder cancer (BCa) is the most common tumor in the urinary system. In 2020, it was estimated that there will be more than 80,000 cases diagnosed in the United States (1). About 75% of bladder cancer patients belong to non-muscle invasive bladder cancer (NMIBC). Transurethral resection of bladder tumor (TURBT) followed by intravesical Bacille Calmette–Guérin (BCG) is the main therapy for high-risk NMIBC (2). The mechanism of intravesical BCG treatment is the immunity it offers after inflammatory response: after the attachment of BCG, innate immunity is induced, which further creates a cytokine milieu, attracting cellular response and triggering tumor-specific immunity (3).

As the age range of patients with a high risk for developing BCa is similar to that of cardiovascular diseases, clinicians often encounter patients taking cardiovascular drugs during the same time of BCG treatment. Some *in vitro* experiments indicated that certain cardiovascular drugs would affect the immune situation and reduce the efficacy of BCG. However, clinical studies showed a conflicting result. Hoffmann et al. demonstrated that breaking off the statin therapy during BCG therapy might improve the clinical outcome (4). In contrast, Skolarus et al. showed that statin use was not associated with adverse outcomes for patients undergoing BCG treatment for bladder cancer (5). Furthermore, Boorjian et al. demonstrated that the risks of recurrence and progression were higher in patients on warfarin, while the risk of progression was lower in patients on aspirin (6). Inversely, Lipsky et al. showed that FCI (fibrin clot inhibitors) did not substantiate a significant impact on BCG efficacy (7).

Different studies give different results, consequently, we aimed to figure out whether or not common cardiovascular drugs will affect the efficacy of BCG. Therefore, we intended to compare the prognosis of patients who took fibrin clot inhibitors or statins during BCG bladder infusion to the prognosis of patients who did not take these medications.

## Methods

The research protocol was carried out according to the Preferred reporting items for systematic review and meta-analyses (PRISMA) statement (8).

### Search Strategy

We selected related research by searching PubMed, Embase, and the Cochrane Central Search Library in March 2020. The following search formula was used: [Fibrin Clot Inhibitors OR Aspirin OR Warfarin OR Clopidogrel OR Statins] AND [Urinary Bladder (Mesh) OR Bladder] AND [Neoplasms (Mesh) OR Cancer OR Carcinoma OR Tumor] AND [BCG Vaccine (Mesh) OR Bacillus Calmette–Guérin OR Bacillus Calmette Guerin Vaccine OR Calmette Vaccine]. We screened all titles and abstracts and searched the references included in the study one by one.

### Inclusion and Exclusion Criteria

We included all randomized controlled studies, cohort studies, and case–control studies that are in the English language.

At the same time, the articles needed to achieve the following criteria: 1) Containing an experimental group and a control group; 2) Containing information about recurrence rate, progression rate, recurrence-free survival (RFS), progression-free survival (PFS), cancer-specific survival (CSS), and overall survival (OS); 3) Application of FCI or statins in the period of intravesical instillation of BCG; 4) BCG is the drug of the intravesical instillation.

On the other hand, ineligible article types such as review articles, case reports, editorials, letters, and conference abstracts were excluded. In addition, there are some exclusion criteria: 1) Cardiovascular drugs that did not include FCI and statins; 2) Instillation of chemotherapy drugs such as gemcitabine and doxorubicin.

### Data Extraction

All the titles and abstracts were independently filtered by two reviewers. If there were encountered disputes, a third reviewer will be consulted to make decisions. The following information was extracted by reviewers: author, study year, country, study type, age, tumor stage, definition of experiment and control, number of samples, events of experiment and control, survival analysis, follow-up, and duration of BCG treatment. Odds ratio (OR) with 95% CI was used for dichotomous variables. Hazard ratio (HR) with 95% CI was used for survival variables. If not available, HR with 95% CI was computed according to Tierney et al.’s suggestions (9). We defined the application of cardiovascular drugs as the experimental group and no drugs as the control group.

### Statistical Analysis and Quality Assessment

As the included pieces of literature are case–control studies and cohort studies, Newcastle-Ottawa Scale was used to assess quality (10). When the score was 7–9, it was considered a high-quality study. Eventually, eleven studies were classified into high quality.

Review Manager 5.3 (The Cochrane Collaboration, The Nordic Cochrane Centre, Copenhagen) was used to complete the meta-analysis. Statistical heterogeneity was detected by using the X^2^ test with a significance set at p <0.10. The degree of heterogeneity was measured by the value of I-squared (I^2^) (I^2^ <25%: no heterogeneity; I^2^ = 25–50%: moderate heterogeneity; I^2^ >50%: large heterogeneity). When the heterogeneity was large, the random-effects model was applied and if not, the fixed-effects model was chosen. A funnel plot was used to assess publication bias. We conducted a sensitivity analysis by the leave-one-out cross-validation, and to assess the stability of the pooling results, Stata/SE 15.1 software was performed.

## Results

### Characteristics of the Included Studies

**Figure 1:**
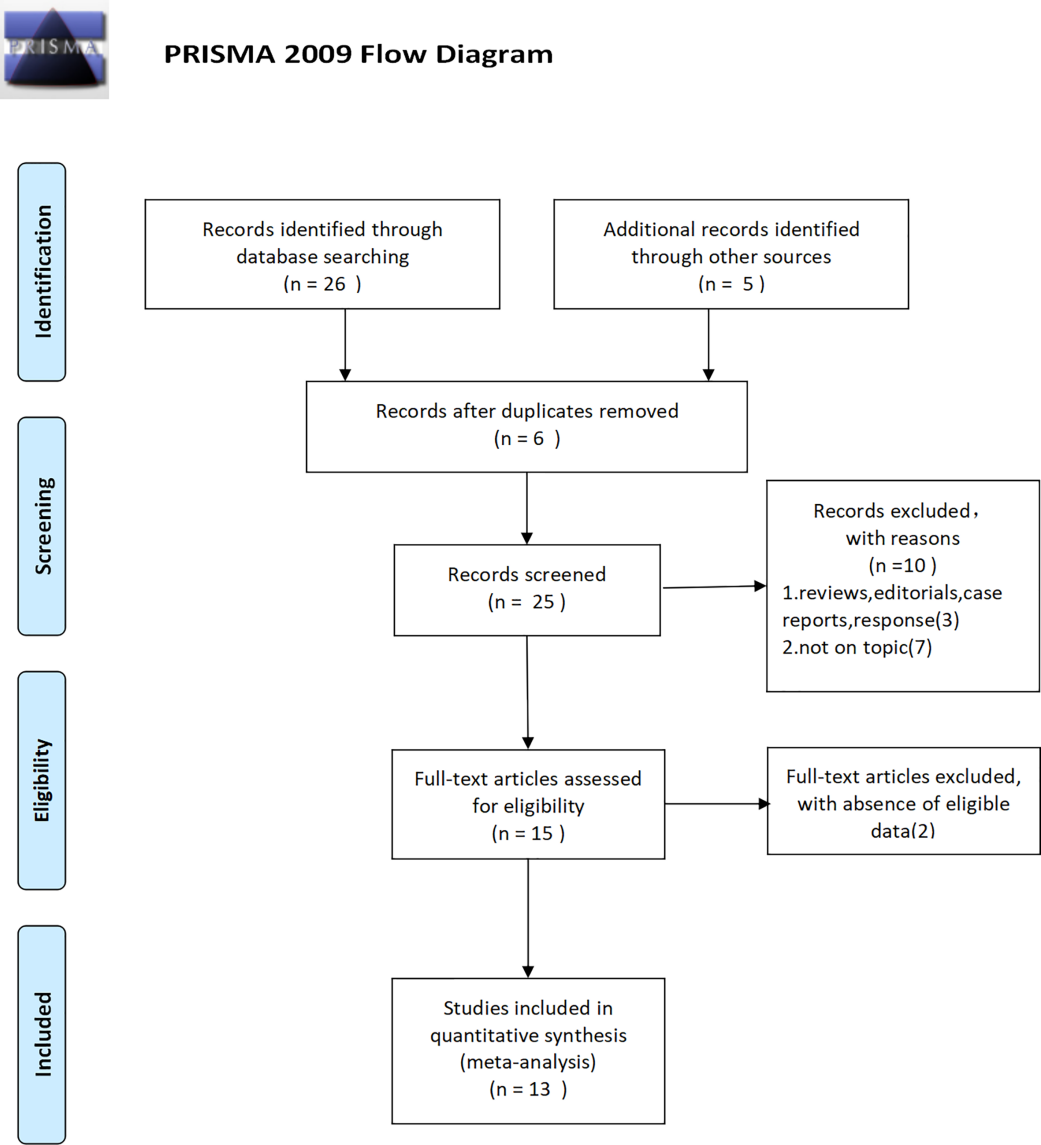
Flow chart of study selection.

Finally, thirteen studies (4–7, 11–19) were included, which contained 3,451 patients. The detailed inclusion process can be seen in (**Figure 1**). Years of these studies ranged from 1990 to 2017; the covered patients were from North America, Europe, Asia, and Oceania. According to the medications that were taken at the time that the research was done, five studies were about statins, seven studies were concerning fibrin clot inhibitors, and one study applied both of them. The comprehensive characteristics of the eligible articles were listed in **Table 1**.

**Table 1:** Comprehensive characteristics of the included patients.

Author	Year	Average age (E/C)	Tumor Stage (E/C)	FCI/Statins	Sample size (E/C)	Follow up (month) (E/C)	Duration of BCG	Endpoints	NOS
Paul Hoffmann	2006	70/63	NA	Statins	19/65	37/50	NA	Progression rate, PFS	7
M’LISS A. HUDSON	1990	NA	Ta/T1/Tis: 29/120 ≥T2:0/0	FCI	29/120	26.5 ± 11.0/ 29.8 ± 13.1	6–12 weeks	Recurrence rate, RFS	7
Ryan K. Berglund	2008	69/65	Ta/T1/Tis: 123/376 ≥T2:5/33	Statins	245/707	51.6	NA	Recurrence rate, RFS	7
Joseph J. Crivelli	2013	65.2/65	Ta/T1/Tis: 341/776 ≥T2:0/0	Statins	341/776	62.7	I: 6 weeks M: 36 months	Recurrence rate, Progression rate, RFS, PFS, CSS, OS	8
Stephen A. Boorjian	2009	69/65	Ta/T1/Tis: 152/480 ≥T2:5/31	FCI	221/686	50.4	6 weeks	Recurrence rate, RFS	7
Ted A Skolarus	2009	68.9/68.1	Ta/T1/Tis: 43/47 ≥T2:0/0	Statins	43/47	56.4 ± 36.0/ 66 ± 38.4	6 weeks	Progression rate, PFS, CSS, OS	8
Jason R. Gee	2008	72/63	Ta/T1/Tis: 20/23 ≥T2:0/0	FCI	20/23	66	NA	Recurrence rate, Progression rate, RFS, PFS	7
Michael J. Lipsky	2013	74/68	Ta/T1/Tis: 89/125 ≥T2:0/0	FCI	89/125	44.1	I: 6 weeks M: 36 months	Recurrence rate, RFS	7
Ashish M. Kamat	2007	NA	NA	Statins	39/117	56	NA	Recurrence rate, Progression rate	5
R.A Gardiner	1993	69/70	Ta/T1/Tis: 9/36 ≥T2:0/0	FCI	9/36	20	6 weeks	Recurrence rate, RFS	7
JA. Witjes	1993	NA	Ta/T1/Tis: 42/141 ≥T2:0/0	FCI	42/141	54	6 weeks	Recurrence rate, RFS	6
Rupesh Gupta	2017	55/58	Ta/T1/Tis: 15/88 ≥T2:0/0	FCI	15/88	10.8	I: 6 weeks M: 18 months	Recurrence rate, Progression rate, RFS, PFS	8
Nirmish Singla	2016	NA	Ta/T1/Tis: 64/99 62/99 ≥T2:0/0	Statins & FCI	64/99 62/99	31.4	I: 6 weeks M: 18 months	RFS, PFS, CSS, OS	7

### Cardiovascular Drugs’ Effect on Recurrence and Progression

**Figure 2:**
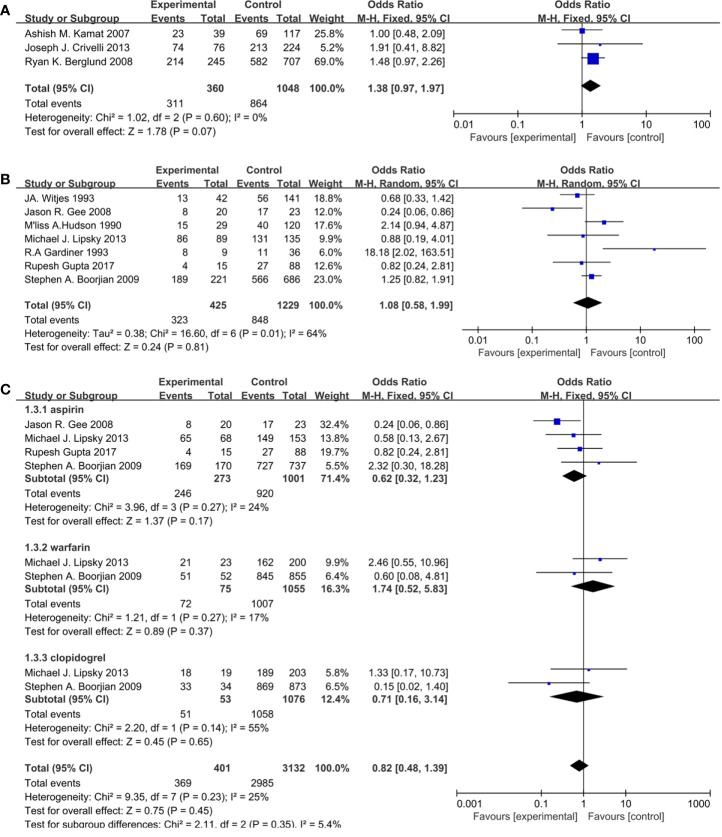
Forest plot for cardiovascular drugs’ effect on recurrence: **(A)** Statins, **(B)** fibrin clot inhibitors, **(C)** subgroup analysis of fibrin clot inhibitors (aspirin, warfarin, and clopidogrel).

The results in the paragraph are dichotomous variables. Ten studies containing 3,062 patients reported cardiovascular drugs’ effect on recurrence. Three studies (**Figure 2A**) applying statins (OR =1.38; 95% CI, 0.97 to 1.97; *p* = 0.07) and seven studies (**Figure 2B**) applying fibrin clot inhibitors (OR = 1.08; 95%CI, 0.58 to 1.99; *p* = 0.81) showed that there was no significant difference between the two groups on recurrence. With no heterogeneity (I^2^ = 0%; p = 0.60), a fixed-effect model was used in statins. With large heterogeneity (I^2^ = 64%; p = 0.01), a random-effect model was used in fibrin clot inhibitors. A subgroup analysis on different fibrin clot inhibitors demonstrated that aspirin (OR = 0.62; 95% CI, 0.32 to 1.23; *p* = 0.17), warfarin (OR = 1.74; 95% CI, 0.52 to 5.83; *p* = 0.37), and clopidogrel (OR = 0.71; 95% CI, 0.16 to 3.14; *p* = 0.65) did not affect the efficacy of BCG on recurrence (**Figure 2C**).

**Figure 3:**
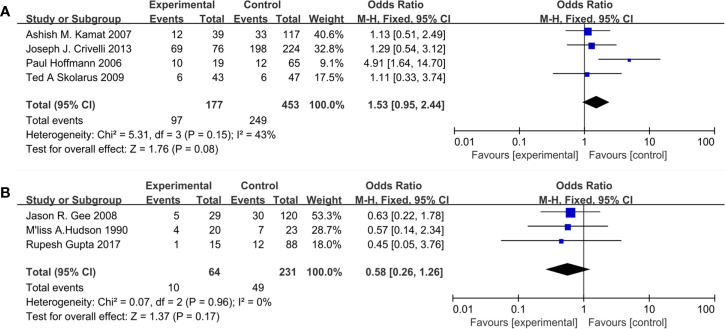
Forest plot for cardiovascular drugs’ effect on progression: **(A)** Statins, **(B)** fibrin clot inhibitors.

Seven studies included 925 patients reporting cardiovascular drugs’ effect on progression. In details, four studies (**Figure 3A**) applying statins (OR = 1.53; 95%CI, 0.95 to 2.44; *p* = 0.08), and the other three studies (**Figure 3B**) applying fibrin clot inhibitors (OR = 0.58; 95% CI, 0.26 to 1.26; *p* = 0.17) showed that there were no significant differences between the two groups on progression. With no heterogeneity in statins (I^2^ = 43%; p = 0.15) and fibrin clot inhibitors (I^2^ = 0%; p = 0.96), a fixed-effect model was used in both of them.

### Cardiovascular Drugs’ Effect on RFS and PFS

**Figure 4:**
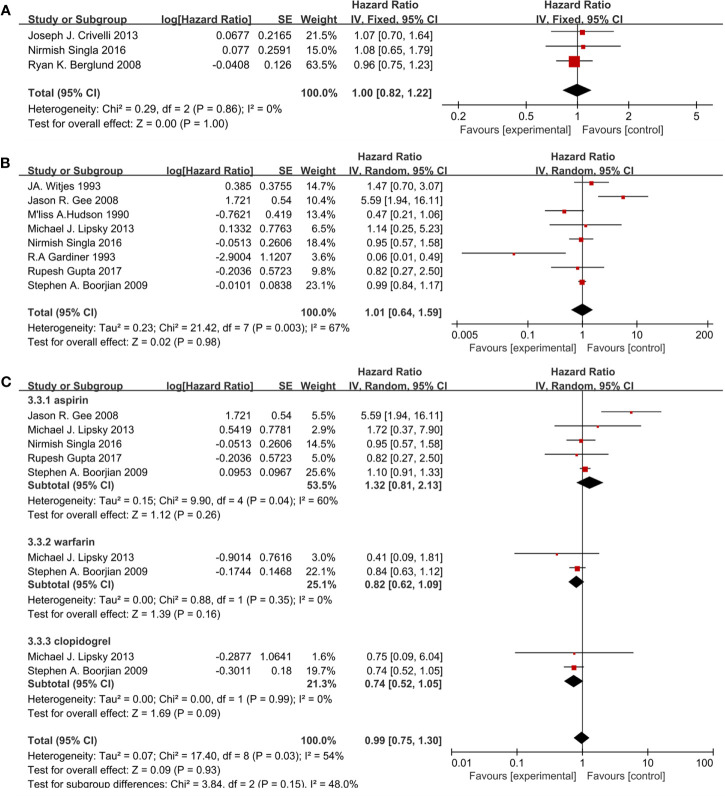
Forest plot for cardiovascular drugs’ effect on RFS: **(A)** Statins, **(B)** fibrin clot inhibitors, **(C)** subgroup analysis of fibrin clot inhibitors (aspirin, warfarin, and clopidogrel).

Eleven studies included 3,121 patients reporting cardiovascular drugs’ effect on RFS. Three studies (**Figure 4A**) applying statins (HR = 1.00; 95%CI, 0.82 to 1.22; *p* = 1.00) and the other eight studies (**Figure 4B**) applying fibrin clot inhibitors (HR = 1.01; 95% CI, 0.64 to 1.59; *p* = 0.98) showed that there were no significant differences between the two groups on RFS. With no heterogeneity in statins (I^2^ = 0%; p = 0.86), a fixed-effect model was used in statins. With no heterogeneity (I^2^ = 67%; p = 0.003), a random-effect model was used in fibrin clot inhibitors. A subgroup analysis on different fibrin clot inhibitors demonstrated that aspirin (HR = 1.32; 95% CI, 0.81 to 2.13; *p* = 0.26), warfarin (HR = 0.82; 95% CI, 0.62 to 1.09; *p* = 0.16) and clopidogrel (HR = 0.74; 95% CI, 0.52 to 1.05; *p* = 0.09) did not affect the efficacy of BCG on RFS (**Figure 4C**).

**Figure 5:**
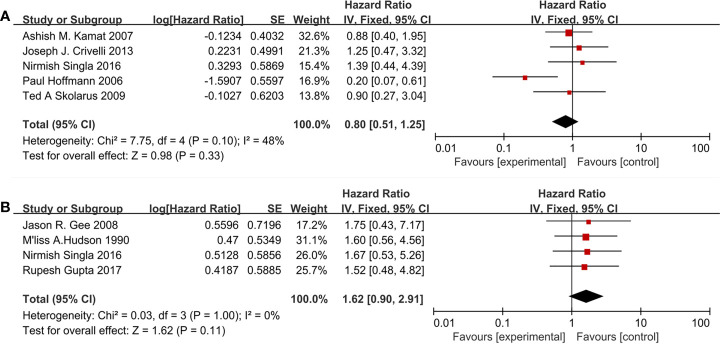
Forest plot for cardiovascular drugs’ effect on PFS: **(A)** Statins, **(B)** fibrin clot inhibitors.

Nine studies included 1,150 patients reporting cardiovascular drugs’ effect on PFS. Five studies (**Figure 5A**) applying statins (HR = 0.79; 95% CI, 0.41 to 1.49; *p* = 0.46) and the other four studies (**Figure 5B**) applying fibrin clot inhibitors (HR = 1.62; 95%CI, 0.90 to 2.91; *p* = 0.11) showed that there were no significant differences between the two groups on PFS. With moderate heterogeneity in statins (I^2^ = 48%; p = 0.10), a fixed-effect model was used in statins. With no heterogeneity (I^2^ = 0%; p = 1.00), a fixed-effect model was used in fibrin clot inhibitors.

### Cardiovascular Drugs’ Effect on CSS and OS

**Figure 6:**
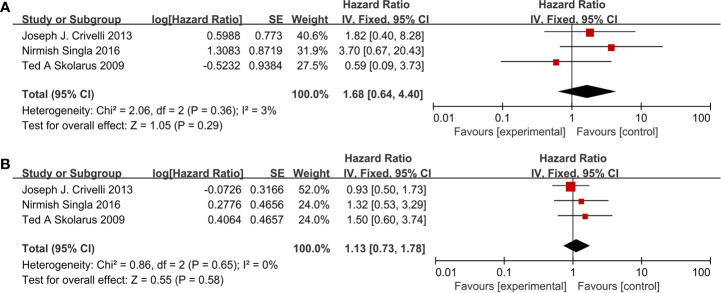
Forest plot for cardiovascular drugs’ effect on CSS and OS: **(A)** CSS, **(B)** OS.

Three studies (**Figure 6A**) included 553 patients reporting cardiovascular drugs’ effect on CSS. All of them applying statins (HR = 1.68; 95% CI, 0.64 to 4.40; *p* = 0.29) showed that there was no significant difference between the two groups on CSS. With no heterogeneity (I^2^ = 3%; p = 0.36), a fixed-effect model was used.

Three studies (**Figure 6B**) included 553 patients reporting cardiovascular drugs’ effect on OS. All of them applying statins (HR = 1.13; 95% CI, 0.73 to 1.78; *p* = 0.58) showed that there was no significant difference between the two groups on OS. With no heterogeneity (I^2^ = 0%; p = 0.65), a fixed-effect model was used.

### Sensitivity Analysis and Publication Bias

In order to assess the stability of results, we conducted leave-one-out cross-validation (**Supplementary Figures 1, 2**) to test the outcomes. The results showed that the overall OR and HR did not change significantly after excluding the studies with heterogeneity.

Furthermore, funnel plots (**Supplementary Figures 3, 4**) were used to evaluate the risk of publication bias, and some asymmetries were found in the result which meant a publication bias did exist.

## Discussion

It has been almost forty years since clinicians have been applying intravesical BCG infusion to reduce the recurrence and progression of bladder cancer. In 1980, Lamm et al. (20) first reported that BCG intravesical therapy following TURBT is better than TURBT only. However, the anti-tumor mechanism of BCG had not been well clarified.

An up-to-date review indicated that BCG affected on anti-tumor procedure by inducing inflammatory cytokines and immune cells into bladder cancer tissues (21). Immune cells including natural killer cells, CD4+/CD8+ lymphocytes, macrophages, granulocytes, and dendritic cells are involved in BCG anti-tumor process (22). Cytokines include IL-1, IL-2, IL-5, IL-6, IL-8, IL-10, IL-12, IL-18, TNF, IFN-*γ* and granulocyte–macrophage colony-stimulating factor (GM-CSF) are involved in BCG anti-tumor process (23).

In our analysis, taking statins during BCG immunotherapy had no effects on patients’ prognosis—RFS and PFS. Theoretically, statins inhibited the immune system in some ways. For example, statins have shown the capability of attenuating the chronic inflammation associated with atherosclerosis (24). It was suggested that statins reduce inflammation by favoring T-helper-2 cell responses over T-helper-1 responses and by up-regulating regulatory T cells (25, 26). However, except for Paul Hoffman’s study, most of the included studies (5, 11, 12, 16, 18) indicated that there was no significant difference in prognosis between the patients taking or not taking statins. For instance, Ryan K. Berglund’s research (11), the largest sample size study which included 245 statin users and 707 control patients, showed similar outcomes on recurrence and RFS between the two groups. From our point of view, the patients in clinical research studies resulted in contradictory outcomes compared to fundamental research studies. For example, statin users were more likely to undergo detections and interventions without delay, which could lead to a better prognosis.

In our results, fibrin clot inhibitors (aspirin, clopidogrel, and warfarin) also did not affect the prognosis of patients—RFS and PFS. On the one hand, some researchers reported that fibrin clot inhibitors would reduce extracellular matrix protein which mediates BCG attachment to the region of urothelial disruption which lessened the effect of BCG (27, 28). On the other hand, fibrin clot inhibitors caused the interruption of the coagulation cascade by local or systemic anticoagulation, preventing the adhesion and implantation of tumor cells (27, 28). When we conducted subgroup analysis according to different fibrin clot inhibitors (aspirin, clopidogrel, and warfarin), the results of subgroup analysis were the same as the comprehensive outcomes. However, Boorjian et al. considered that aspirin and warfarin had the opposite effect on BCG therapy——the risks of recurrence and progression were higher in patients on warfarin, while the risk of progression was lower in patients on aspirin (6). We hold the opinion that aspirin repressing Cox enzymes, especially Cox-2, would increase the production of interleukin-12 after BCG therapy, which could enhance the efficacy of BCG. However, warfarin lacked this underlying mechanism.

The reasons for the differences we found while analyzing are the following: First of all, the studies that indicated positive results have the same feature: small sample size. Their experimental groups just contained nine, nineteen, and twenty patients, respectively. Moreover, the included studies lacked dose-related data, so the confounding factors of drug dosage in the pooled analysis could not be assessed.

There were some limitations in our meta-analysis. Above all, all eligible studies were not randomized controlled trials. The lack of random sequence generation and blinding of participants might lead to various biases. Secondly, some included pieces of literature did not give the duration and dosage of the BCG therapy taken. Consequently, we cannot analyze the effect of FCI/statins during the induction period and the maintenance period. Finally, several HRs are calculated based on the data derived from the survival curve, which might cause deviations.

## Conclusion

The present study shows that the application of fibrin clot inhibitors and statins does not influence the efficacy of BCG on oncological prognosis. Consequently, we do not need to stop using them or change to other drugs during intravesical BCG treatment. However, large-scale multi-center prospective research is still needed to confirm the above conclusions.

## Author Contributions

Conception and design: XZ, ZC, and JC. Administrative support: XZ and JC. Provision of study materials or patients: JH and ZC. Collection and assembly of data: ZC and JH. Data analysis and interpretation: ZC, JH, HL, ZY, and DQ. Manuscript writing: ZC and JH. Language edit: BO. All authors contributed to the article and approved the submitted version.

## Funding

This work was supported by the National Natural Science Foundation of China (81873626, 81902592), Hunan Province Key R&D Program (2019SK2202), and Xiangya Hospital Youth Fund (2018Q09).

## Conflict of Interest

The authors declare that the research was conducted in the absence of any commercial or financial relationships that could be construed as a potential conflict of interest.

## Supplementary Material

Supplementary Figure 1 Sensitivity analysis for dichotomous variable: **(A)** Recurrence in statins, **(B)** recurrence in fibrin clot inhibitors, **(C)** progression in statins, **(D)** progression in fibrin clot inhibitors.

Supplementary Figure 2 Sensitivity analysis for survival variable: **(A)** RFS in statins, **(B)** RFS in fibrin clot inhibitors, **(C)** PFS in statins, **(D)** PFS in fibrin clot inhibitors, **(E)** CSS, **(F)** OS.

Supplementary Figure 3 Funnel plot for dichotomous variable: **(A)** Recurrence in statins, **(B)** recurrence in fibrin clot inhibitors, **(C)** progression in statins, **(D)** progression in fibrin clot inhibitors.

Supplementary Figure 4 Funnel plot for survival variable: **(A)** RFS in statins, **(B)** RFS in fibrin clot inhibitors, **(C)** PFS in statins, **(D)** PFS in fibrin clot inhibitors, **(E)** CSS, **(F)** OS.
