# Supplementary figures and images for: Effects of Fibrin Clot Inhibitors and Statins on the Intravesical Bacille Calmette–Guérin Therapy for Bladder Cancer: A Systematic Review and Meta-Analysis
Source: Front Oncol. 2021 Jun 21;11:614041. doi: 10.3389/fonc.2021.614041 (PMC8256157; doi:10.3389/fonc.2021.614041)

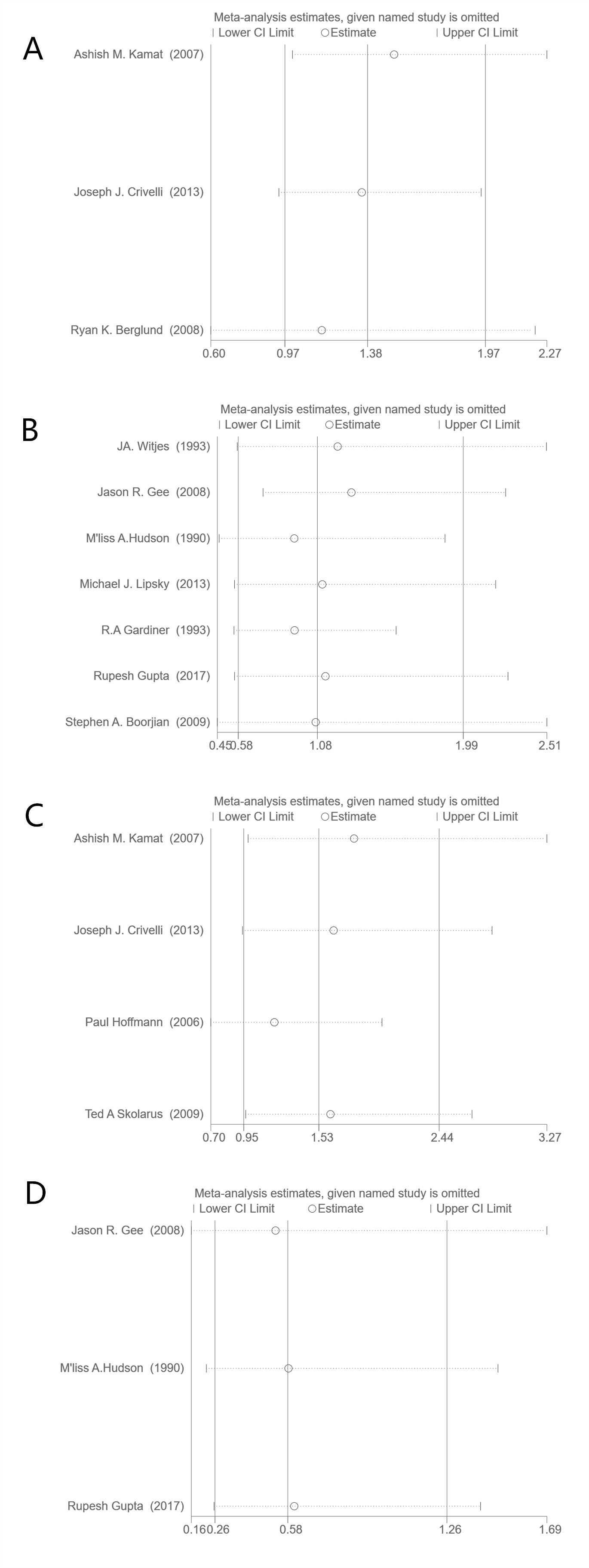

Supplement: Supplementary Figure 1 — Sensitivity analysis for dichotomous variable: (A) Recurrence in statins, (B) recurrence in fibrin clot inhibitors, (C) progression in statins, (D) progression in fibrin clot inhibitors. [file Image_1.tif]

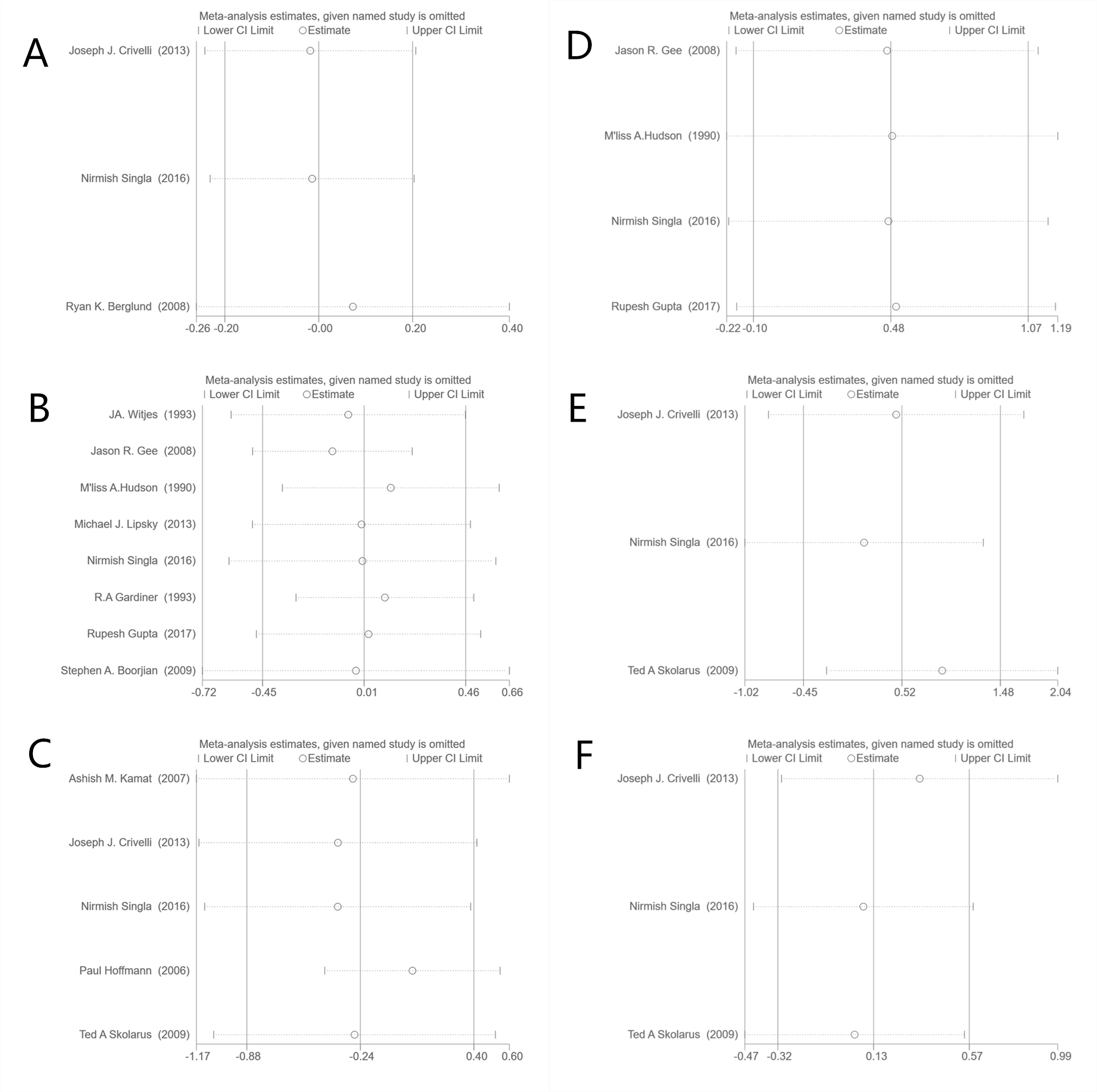

Supplement: Supplementary Figure 2 — Sensitivity analysis for survival variable: (A) RFS in statins, (B) RFS in fibrin clot inhibitors, (C) PFS in statins, (D) PFS in fibrin clot inhibitors, (E) CSS, (F) OS. [file Image_2.tif]

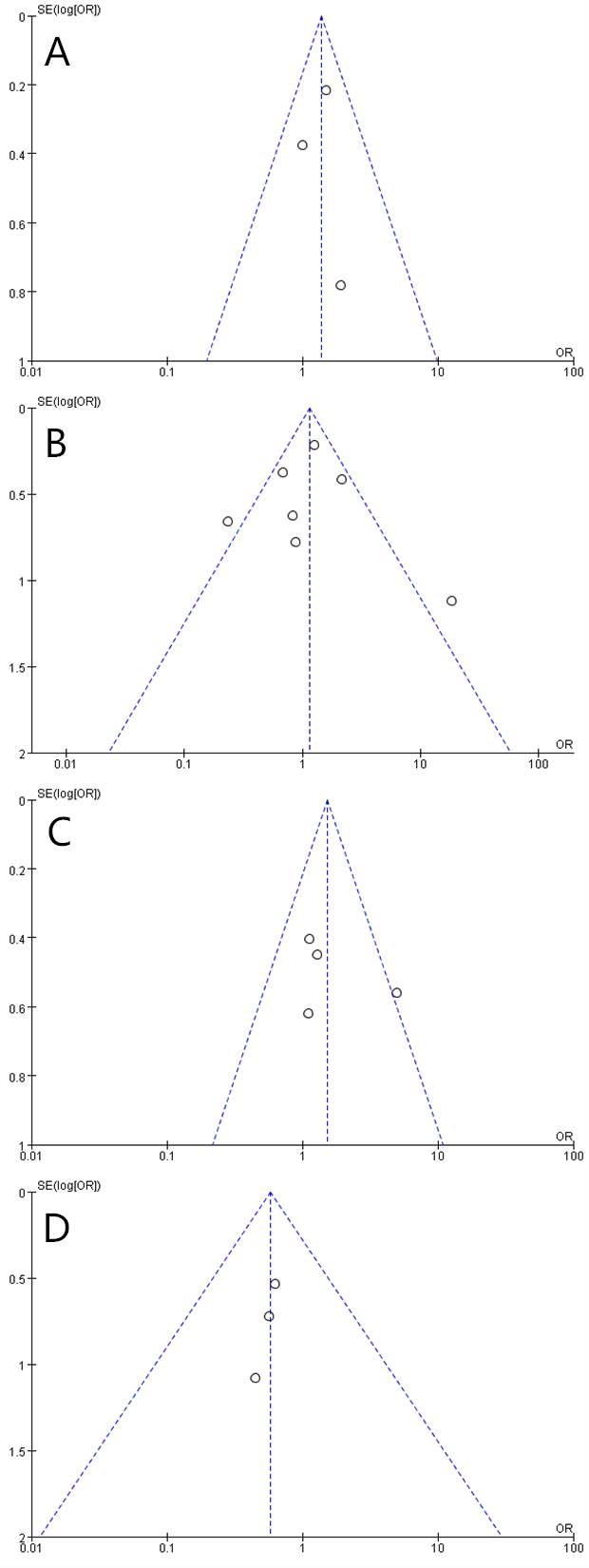

Supplement: Supplementary Figure 3 — Funnel plot for dichotomous variable: (A) Recurrence in statins, (B) recurrence in fibrin clot inhibitors, (C) progression in statins, (D) progression in fibrin clot inhibitors. [file Image_3.tif]

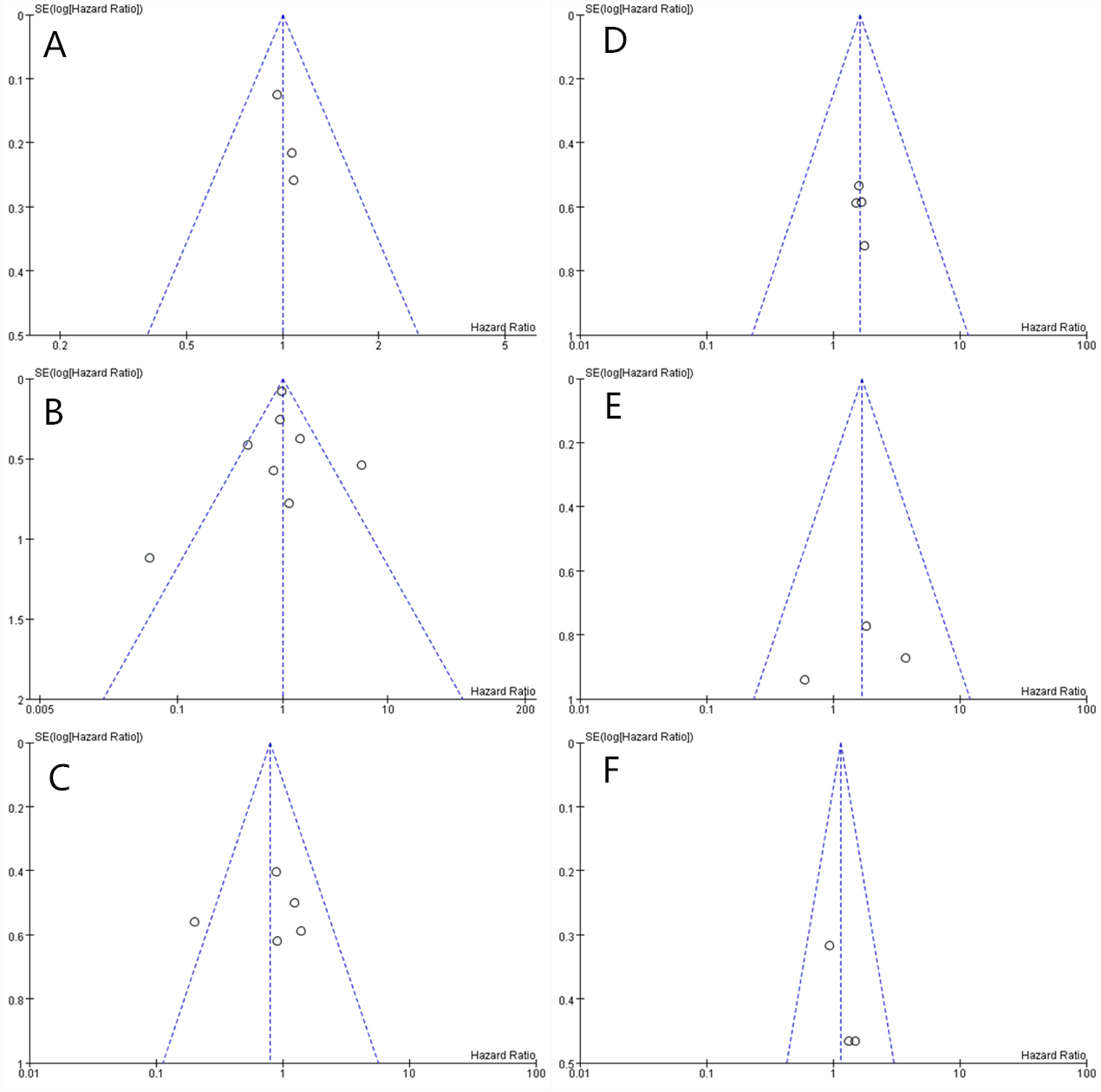

Supplement: Supplementary Figure 4 — Funnel plot for survival variable: (A) RFS in statins, (B) RFS in fibrin clot inhibitors, (C) PFS in statins, (D) PFS in fibrin clot inhibitors, (E) CSS, (F) OS. [file Image_4.tif]
